# Supplementary material for: Blood DNA methylation profiling identifies cathepsin Z dysregulation in pulmonary arterial hypertension
Source: Nat Commun. 2024 Jan 6;15:330. doi: 10.1038/s41467-023-44683-0 (PMC10771427; doi:10.1038/s41467-023-44683-0)
Supplement: Supplementary file 1 — Supplementary Information [file 41467_2023_44683_MOESM1_ESM.pdf]

Supplementary Materials for

Blood DNA Methylation Profiling Identifies Cathepsin Z Dysregulation in Pulmonary Arterial Hypertension

Anna Ulrich 1*, Yukyee Wu 2*, Harmen Draisma 1,3, John Wharton 2, Emilia M Swietlik 4, Inês Cebola 3, Eleni Vasilaki 2, Zhanna Balkhiyarova 1,3,5 Marjo-Riitta Jarvelin 6,7,8,9 Juha Auvinen 7, Karl-Heinz Herzig 10,11, J. Gerry Coghlan 12, James Lordan 13, Colin Church 14, Luke S Howard 2, Joanna Pepke-Zaba 15, Mark Toshner 4, Stephen J Wort 2, 16, David G Kiely 17, 18, 19, Robin Condliffe 17, 18, Allan Lawrie 2,17, Stefan Gräf 4, 20, Nicholas W Morrell 4, Martin R Wilkins 2, Inga Prokopenko 1†, Christopher J Rhodes 2†

*These authors contributed equally

†These authors jointly supervised this work

	Affiliation(s)	Country
1	Department of Clinical and Experimental Medicine, University of Surrey	United Kingdom
2	National Heart and Lung Institute, Imperial College London	United Kingdom
3	Section of Genetics & Genomics, Department of Metabolism, Digestion and Reproduction, Imperial College London	United Kingdom
4	VPD Heart & Lung Research Institute, University of Cambridge	United Kingdom
5	People-Centred Artificial Intelligence Institute, University of Surrey, Guildford	United Kingdom
6	MRC Centre for Environment and Health, Department of Epidemiology and Biostatistics, School of Public Health, Imperial College London	United Kingdom
7	Center for Life Course Health Research, Faculty of Medicine, University of Oulu	Finland
8	Unit of Primary Care, Oulu University Hospital, Oulu	Finland
9	Department of Life Sciences, College of Health and Life Sciences, Brunel University London	United Kingdom
10	Institute of Biomedicine, Medical Research Center Oulu, Oulu University and Oulu University Hospital, Finland	Finland
11	Department of Pediatric Gastroenterology and Metabolic Diseases, Poznan University of Medical Sciences, Poznan, Poland	Poland
12	University College London	United Kingdom
13	University of Newcastle	United Kingdom
14	Golden Jubilee National Hospital and University of Glasgow	United Kingdom
15	Royal Papworth Hospital, Cambridge	United Kingdom
16	National PH Service, Royal Brompton Hospital, London	United Kingdom
17	Department of Infection, Immunity & Cardiovascular Disease, University of Sheffield	United Kingdom
18	Sheffield Pulmonary Vascular Disease Unit, Royal Hallamshire Hospital, Sheffield	United Kingdom
19	NIHR Biomedical Research Centre Sheffield	United Kingdom
20	NIHR BioResource for Translational Research, Cambridge Biomedical Campus	United Kingdom

Correspondence to: Dr Chris Rhodes, crhodes@ic.ac.uk, Imperial College London, Vascular Section NHLI, Hammersmith Campus, Du Cane Road, LONDON, W12 0NN, United Kingdom

Supplementary Materials

For Supplementary Data, please see the supplemental Excel file provided.

Supplementary Data 1: Study demographics and white blood cell fractions. Age and white blood cell fractions are presented as median (interquartile range). P-values for between-group differences were derived from Chi-squared tests of independence and one-way ANOVA tests for categorical and continuous variables, respectively. Abbreviations: NK = natural killer. * P-values from two-sided paired T-tests between baseine and follow-up values.

Supplementary Data 2: Study demographics, white blood cell fractions and clinical characteristics of baseline and follow-up samples. Abbreviations: NK = natural killer. P-values were derived from two-sided paired T-tests and McNemar tests for continuous and categorical variables, respectively.

Supplementary Data 3: Top CpG markers associated with PAH reaching a significance threshold of $P<10^{-5}$. Three CpG markers reaching the genomic-inflation-factor-adjusted epigenome-wide significance threshold of $P<10^{-7}$ are highlighted. Results are multivariable regression analysis (main EWAS). Effect1 Effect estimate of CpG marker on PAH susceptibility in log(OR) per % increase in methylation. Gene Target gene name from the UCSC database.

Supplementary Data 4: Top three CpG marker regions associated with PAH. CpG markers present in the corresponding regional plot are listed, lead CpG markers are highlighted. Results are multivariable regression analysis (main EWAS). Effect1 Effect estimate of CpG marker on PAH susceptibility in log(OR) per % increase in methylation. Correlation coefficient with lead CpG marker, Spearman`s rank correlation coefficient. P value λ adjusted P-values adjusted for the genomic inflation factor of this study (λ=1.45).

Supplementary Data 5: CpG marker associations with PAH at 16 established PAH genes. CpG marker(s) with Q-values < 0.05 are highlighted. Results are multivariable regression analysis (main EWAS). Effect1 Effect estimate of CpG marker on PAH susceptibility in log(OR) per % increase in methylation. Gene region feature category Gene region feature category describing the CpG position, from UCSC. P-values adjusted for multiple comparisons using the Benjamini & Hochberg method.

Supplementary Data 6: Transcript abundance and CpG methylation associations between the top three CpG markers and genes within the CpG-marker-containing TAD (topologically associating domain). Multivariable regression analysis. Effect2 Effect estimate of the CpG marker on transcript abundance (RNAseq) in beta units per TPM. P value FDR adjusted P-values adjusted for multiple comparisons using the Benjamini & Hochberg method.

Supplementary Data 7 - Log10 fold-changes and associated statistics from whole blood RNA comparisons between PAH and healthy controls from UK PAH Cohort study, and correlations with CpG methylation status of epigenetic regulators. Discovery (n=24 controls versus 120 PAH patients) and validation (another n=24/120) groups are combined for final analysis by edgeR corrected for white blood cells estimated from RNAseq data, age, sex and principal components as described in Rhodes, Otero-Nunez et al AJRCCM 2021. Spearman's Rank correlations performed in all patients with overlapping data.

Supplementary Data 8 - Correlation of CpG profiles with markers of disease severity. Rho values and significance of Spearman's rank tests.

Supplementary Data 9 - Linear regression model for plasma cathepsin Z levels in PAH patients. Plasma proteins used to predict cathepsin Z levels all measured on SomaLogic SomaScan platform as described in Rhodes et al AJRCCM 2022

Supplementary Data 10 - Details of individuals who donated lung tissue for immunohistochemistry.

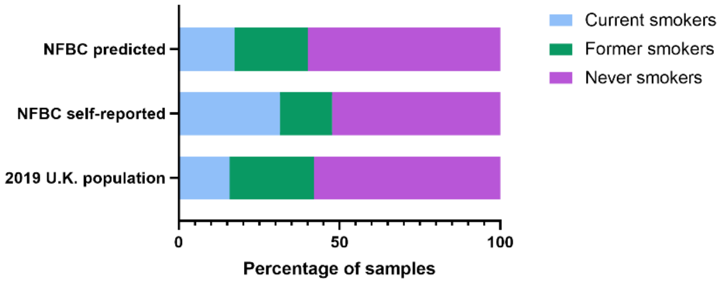

Supplementary Figure 1. Smoking prediction in NFBC1966 using EpiSmokEr compared to self-reported survey data in NFBC1966 and general UK population circa 2019 (source: UK Office for National Statistics [57]).

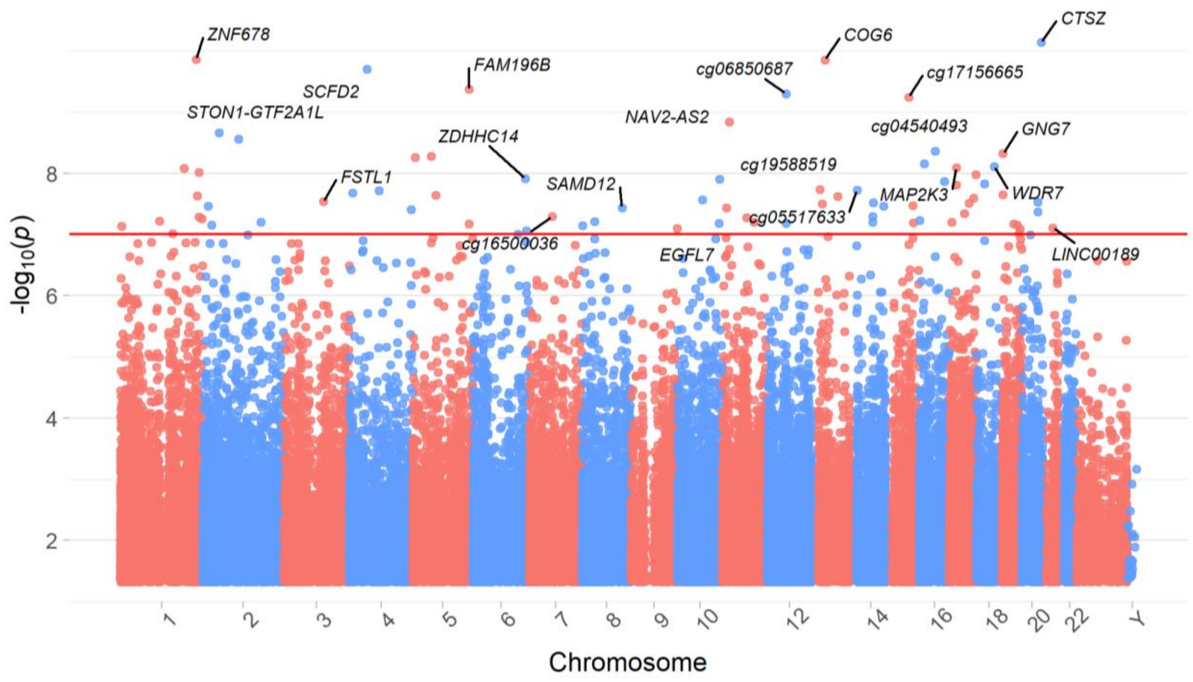

Supplementary Figure 2. Upper: Manhattan plot of epigenome-wide association analysis of PAH. DNAme markers are ordered according to their genomic positions along the x-axis. P-values (without adjustment for genomic inflation) for the CpG marker effect are plotted along the y-axis on the $-\log_{10}$ scale. The red horizontal line corresponds to the epigenome-wide significance threshold of $P<10^{-7}$ with annotated markers reaching this threshold. CpG markers with the lowest P-value in each chromosome are annotated with the name of the nearby gene, where available, or the CpG marker name otherwise.

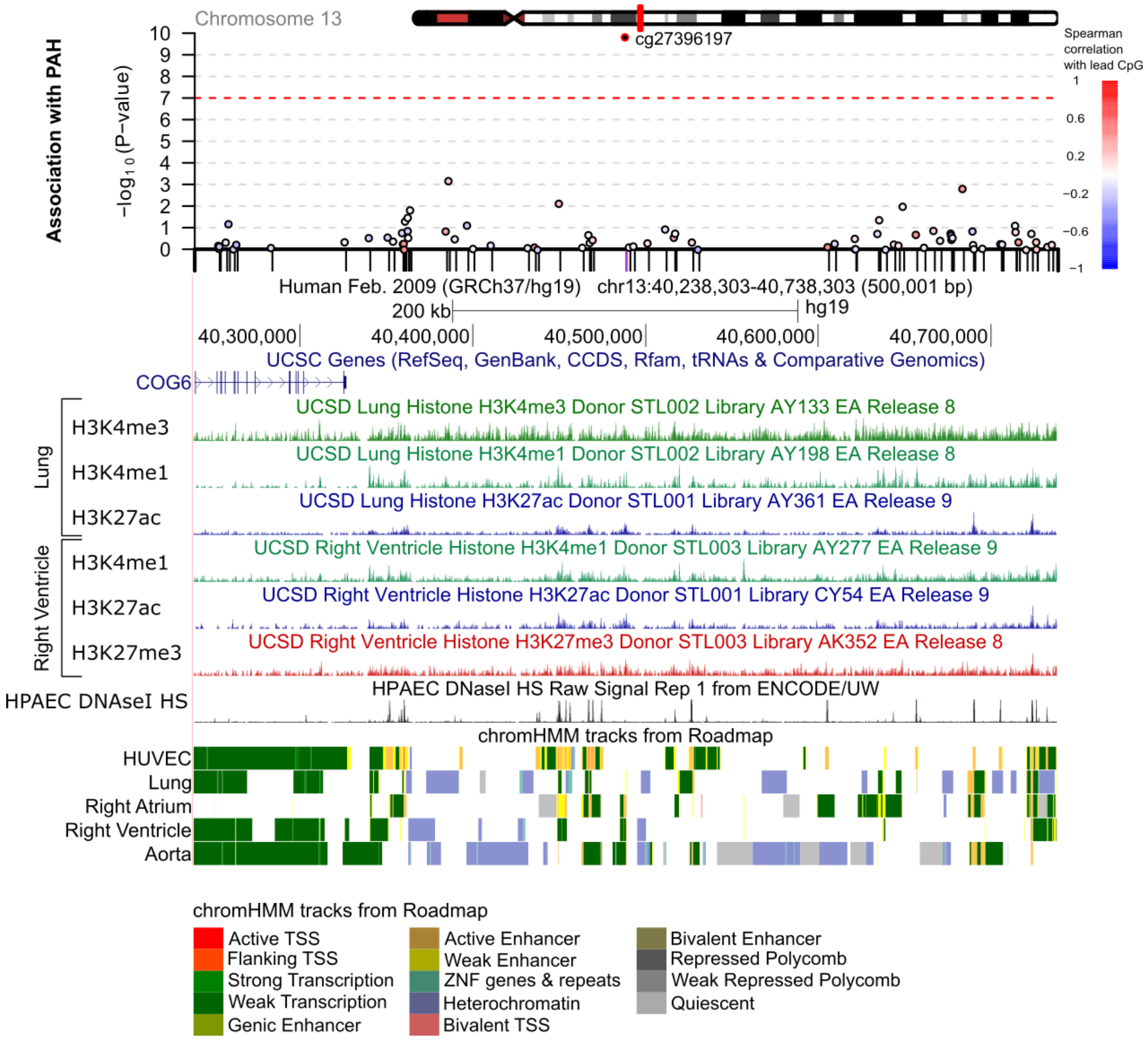

Supplementary Figure 3. Genomic region containing the significant DNAme marker near *COG6* identified by the epigenome-wide association study (EWAS). A 50-kilobase region on either side of the top DNAme marker (cg27396197) is shown. Each circle represents a DNAme marker colored by its correlation value with the top DNAme marker (only markers with significant [P <0.05] Spearman`s rank correlation coefficients are colored; red – positive, blue – negative). Epigenomic data in endothelial cells including pulmonary artery endothelial cells (HPAEC) and human umbilical vein-derived endothelial cells (HUVEC), lung and the right heart indicate areas likely to contain active regulatory regions and promoters. Markers include histone H3 lysine 4 monomethylation (H3K4Me1; often found in enhancers) and trimethylation (H3K4Me3; strongly observed in promoters) and H3 lysine 27 acetylation (H3K27Ac; often found in active regulatory regions). Auxiliary hidden Markov models, which summarise epigenomic data to predict the functional status of genomic regions in different tissues or cells, are shown (chromHMM). Red marks signal active transcription start sites. Annotation data were extracted from the UCSC website [56]. The UCSC Session URL is available in the Supplementary Materials section.

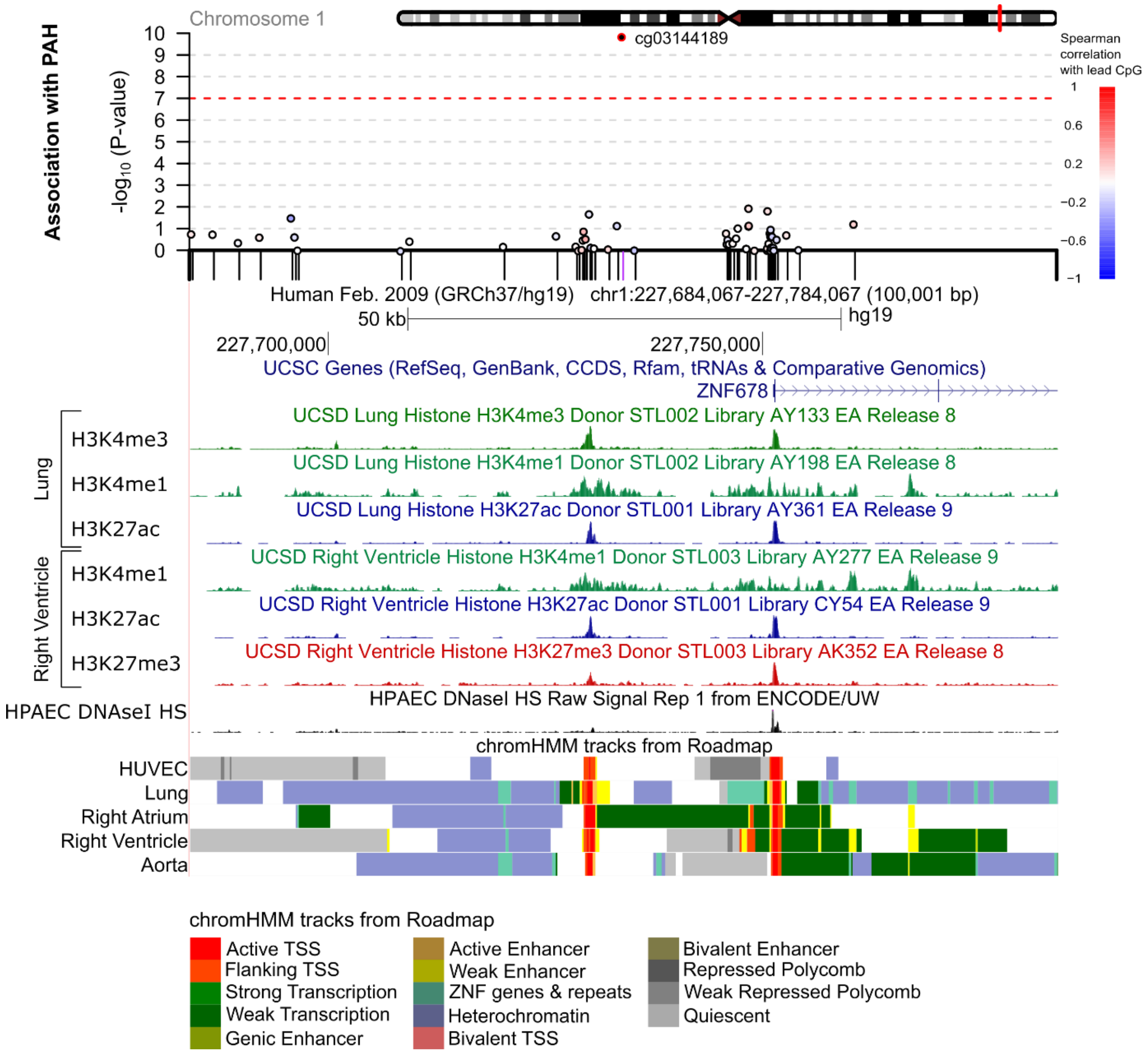

Supplementary Figure 4. Genomic region containing the significant DNAme marker near *ZNF678* identified by the epigenome-wide association study (EWAS). A 50-kilobase region on either side of the top DNAme marker (cg03144189) is shown. Each circle represents a DNAme marker colored by its correlation value with the top DNAme marker (only markers with significant [*P* <0.05] Spearman`s rank correlation coefficients are colored; red-positive, blue-negative). Epigenomic data in endothelial cells including pulmonary artery endothelial cells (HPAEC) and human umbilical vein-derived endothelial cells (HUVEC), lung and the right heart indicate areas likely to contain active regulatory regions and promoters. Markers include histone H3 lysine 4 monomethylation (H3K4Me1; often found in enhancers) and trimethylation (H3K4Me3; strongly observed in promoters) and H3 lysine 27 acetylation (H3K27Ac; often found in active regulatory regions). Auxiliary hidden Markov models, which summarise epigenomic data to predict the functional status of genomic regions in different tissues or cells, are shown (chromHMM). Red marks signal active transcription start sites. Annotation data were extracted from the UCSC website [56]. The UCSC Session URL is available in the Supplementary Materials section.

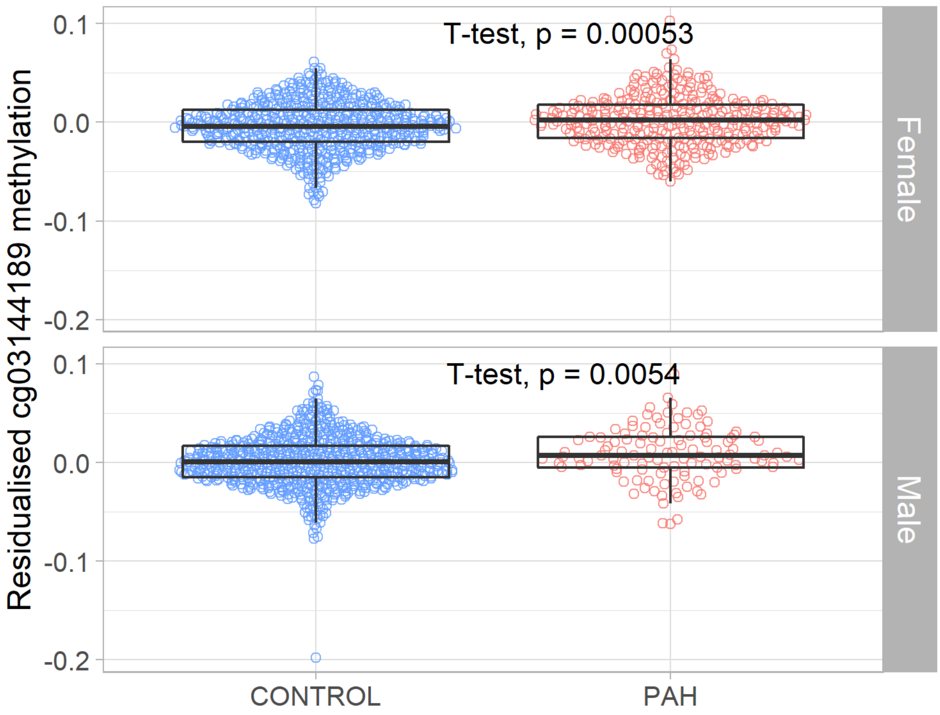
Residualised cg03144189 methylation
T-test, p = 0.00053
T-test, p = 0.0054
0.1
0.0
-0.1
-0.2
Female
Male
CONTROL
PAH

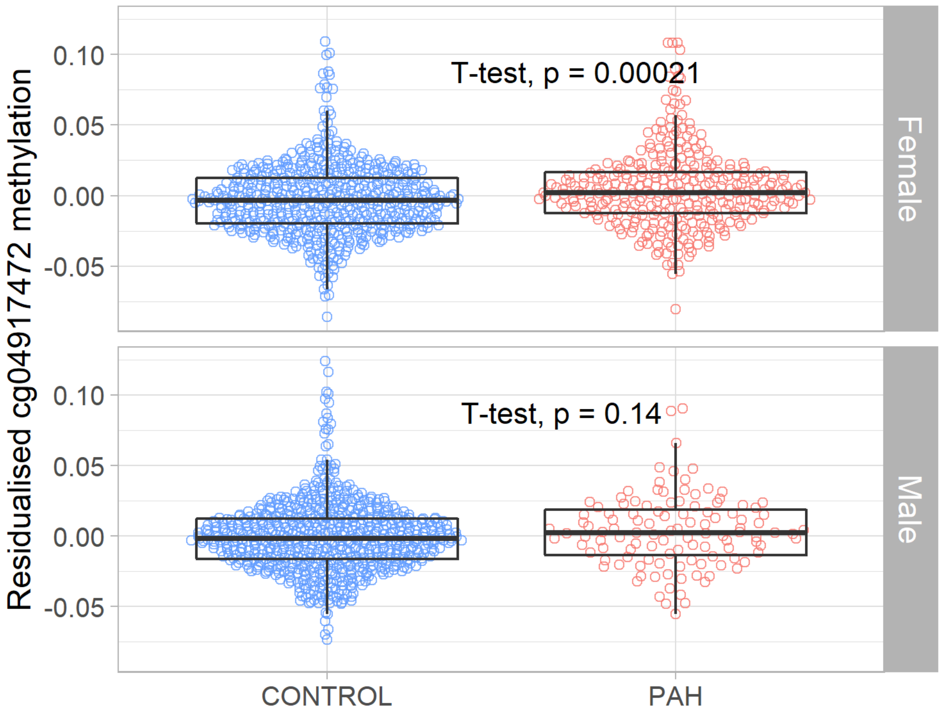
Residualised cg04917472 methylation
T-test, p = 0.00021
T-test, p = 0.14
0.10
0.05
0.00
-0.05
Female
Male
CONTROL
PAH

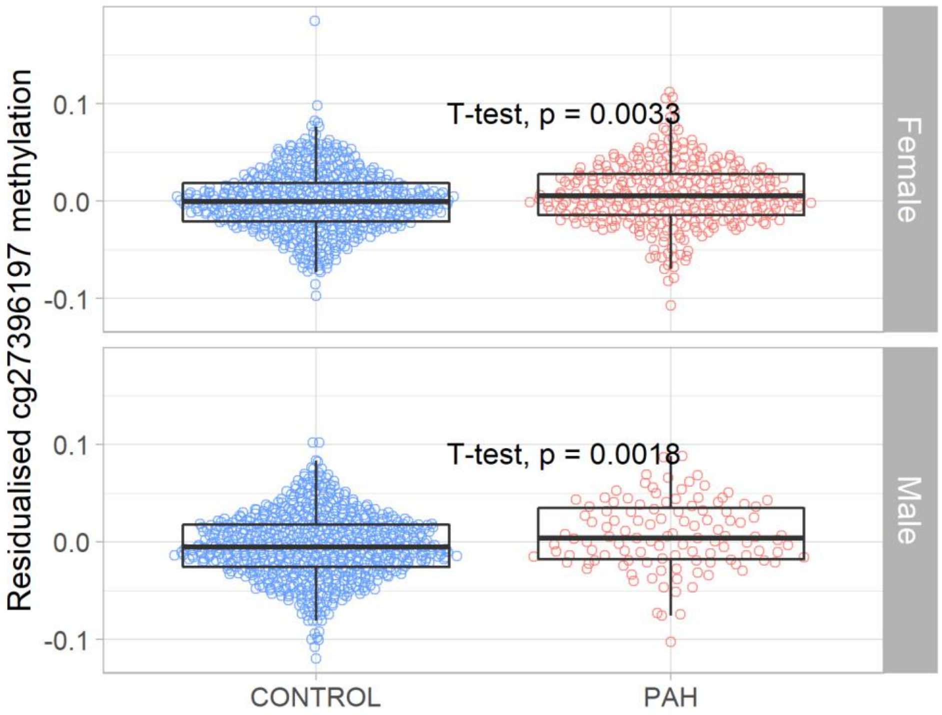

Supplementary Figure 5. Analysis of the three lead CpG sites between controls and PAH individuals in females and male separately. P-values shown on the top are from unpaired T-tests comparing the residuals of DNAme levels - adjusted for the EWAS covariates – between male or female controls and PAH patients.

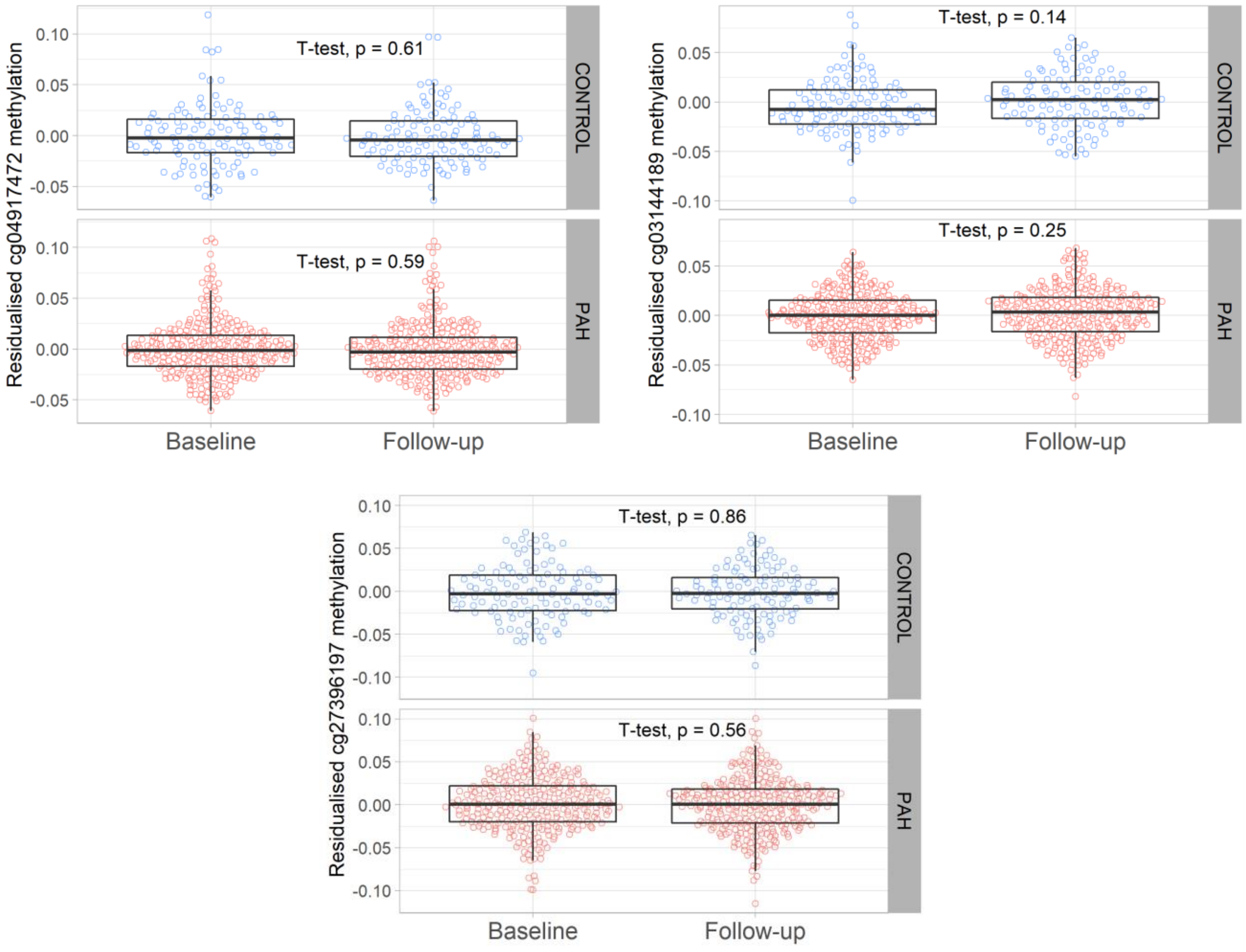

Supplementary Figure 6. Methylation levels of the three PAH-associated DNAme markers in baseline and follow-up samples of PAH and controls from the ADNI cohort. Residuals of CpG methylation levels after adjusting for covariates in the EWAS are plotted along the y-axis. Boxplots show the median, interquartile range (IQR) and whiskers extend to 1.5 times the IQR. P-value from the paired t-test is shown.

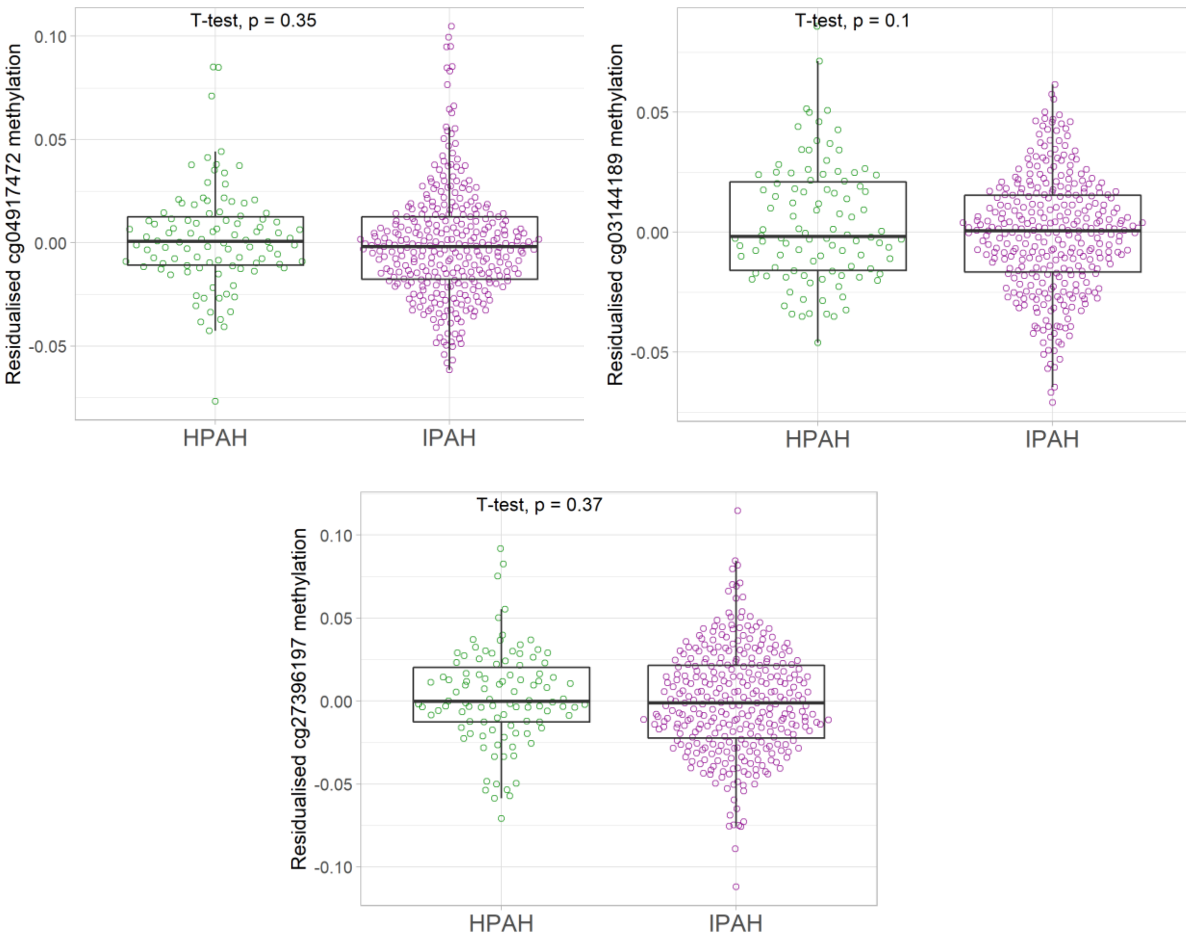

Supplementary Figure 7. Methylation levels of the three PAH-associated DNAme markers in heritable versus idiopathic PAH samples. Residuals of CpG methylation levels after adjusting for covariates in the EWAS are plotted along the y-axis. Boxplots show the median, interquartile range (IQR) and whiskers extend to 1.5 times the IQR. P-value from the unpaired t-test is shown.

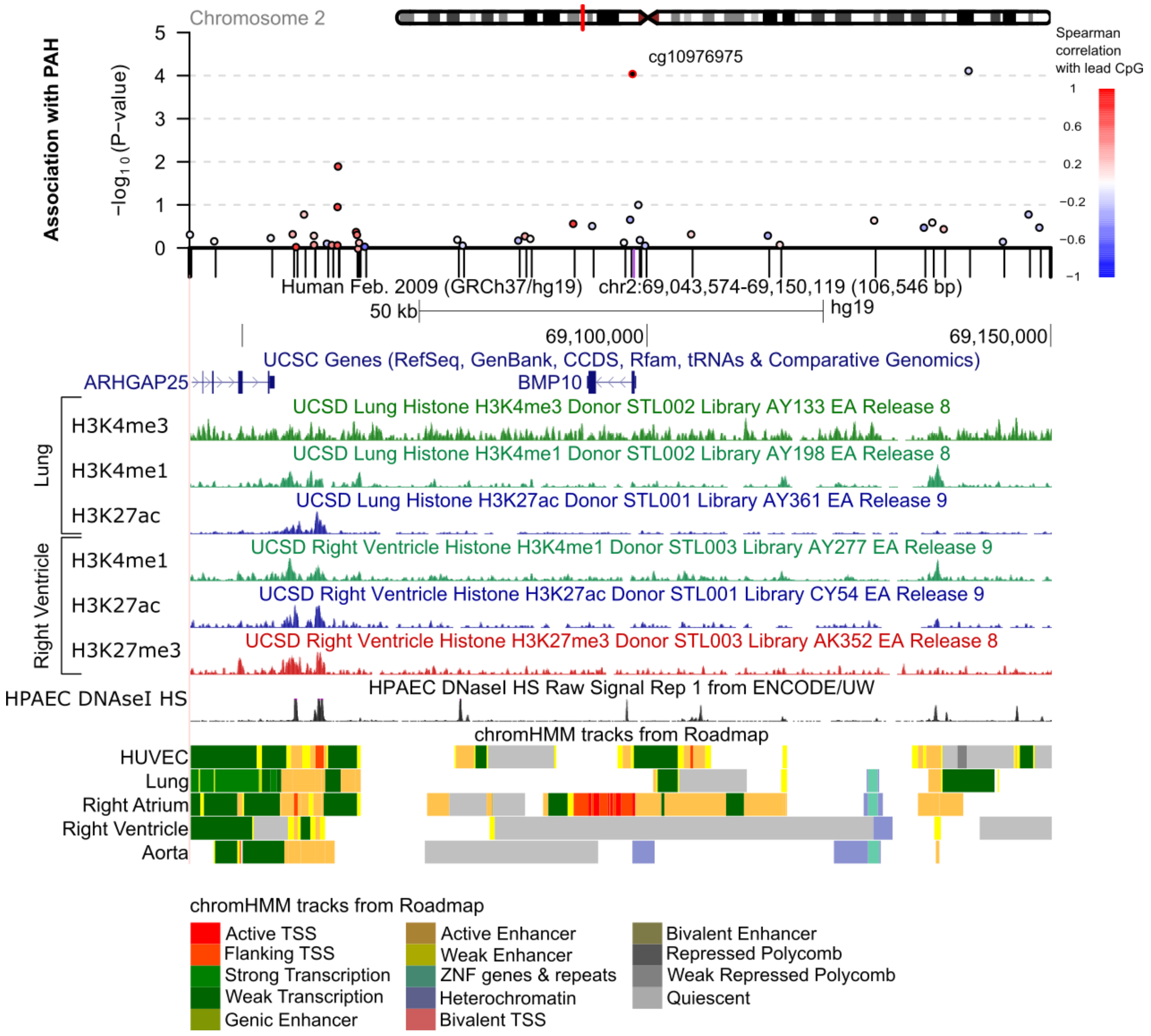

Supplementary Figure 8. Genomic region containing the DNAme marker in the 5' untranslated region of BMP10 identified by the epigenome-wide association study (EWAS). A ~50-kilobase region on either side of the top DNAme marker (cg10976975) is shown. Each circle represents a DNAme marker colored by its correlation value with the top DNAme marker (only markers with significant [P <0.05] Spearman`s rank correlation coefficients are colored; red-positive, blue-negative). Epigenomic data in endothelial cells including pulmonary artery endothelial cells (HPAEC) and human umbilical vein-derivedumbilical vein endothelial cells (HUVEC), lung and the right heart indicate areas likely to contain active regulatory regions and promoters. Markers include histone H3 lysine 4 monomethylation (H3K4Me1; often found in enhancers) and trimethylation (H3K4Me3; strongly observed in promoters) and H3 lysine 27 acetylation (H3K27Ac; often found in active regulatory regions). Auxiliary hidden Markov models, which summarise epigenomic data to predict the functional status of genomic regions in different tissues or cells, are shown (chromHMM). Red marks signal active transcription start sites. Annotation data were extracted from the UCSC website [56]. The UCSC Session URL is available in the Supplementary Materials section.

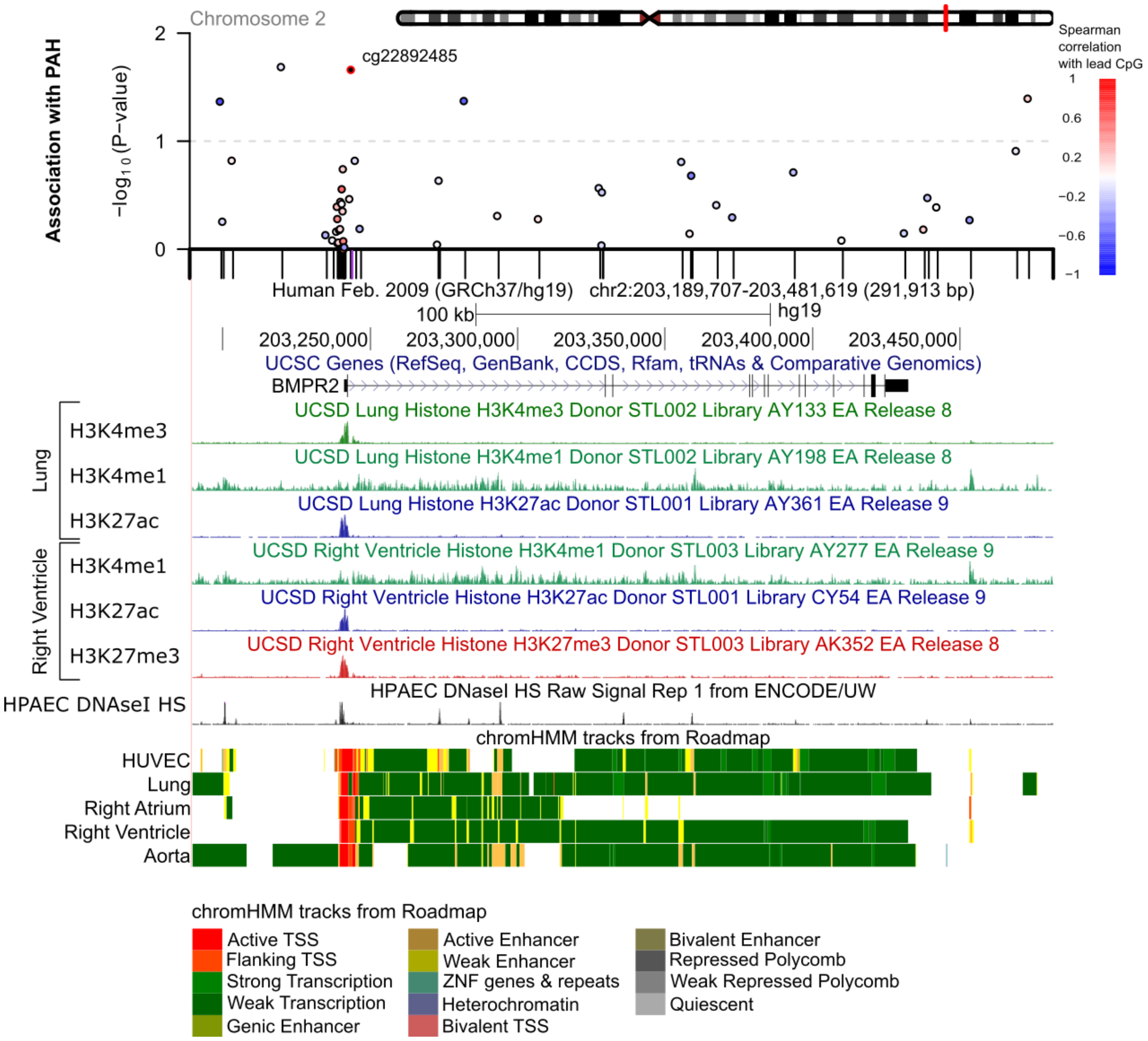

Supplementary Figure 9. Genomic region containing the DNAme markers near *BMPR2*. A 50-kilobase region on either side of the gene is shown. Each circle represents a DNAme marker colored by its correlation value with the top DNAme marker (only markers with significant [*P* <0.05] Spearman`s rank correlation coefficients are colored; red-positive, blue-negative). Epigenomic data in endothelial cells including pulmonary artery endothelial cells (HPAEC) and human umbilical vein-derived endothelial cells (HUVEC), lung and the right heart indicate areas likely to contain active regulatory regions and promoters. Markers include histone H3 lysine 4 monomethylation (H3K4Me1; often found in enhancers) and trimethylation (H3K4Me3; strongly observed in promoters) and H3 lysine 27 acetylation (H3K27Ac; often found in active regulatory regions). Auxiliary hidden Markov models, which summarise epigenomic data to predict the functional status of genomic regions in different tissues or cells, are shown (chromHMM). Red marks signal active transcription start sites. Annotation data were extracted from the UCSC website [56]. The UCSC Session URL is available in the Supplementary Materials section.

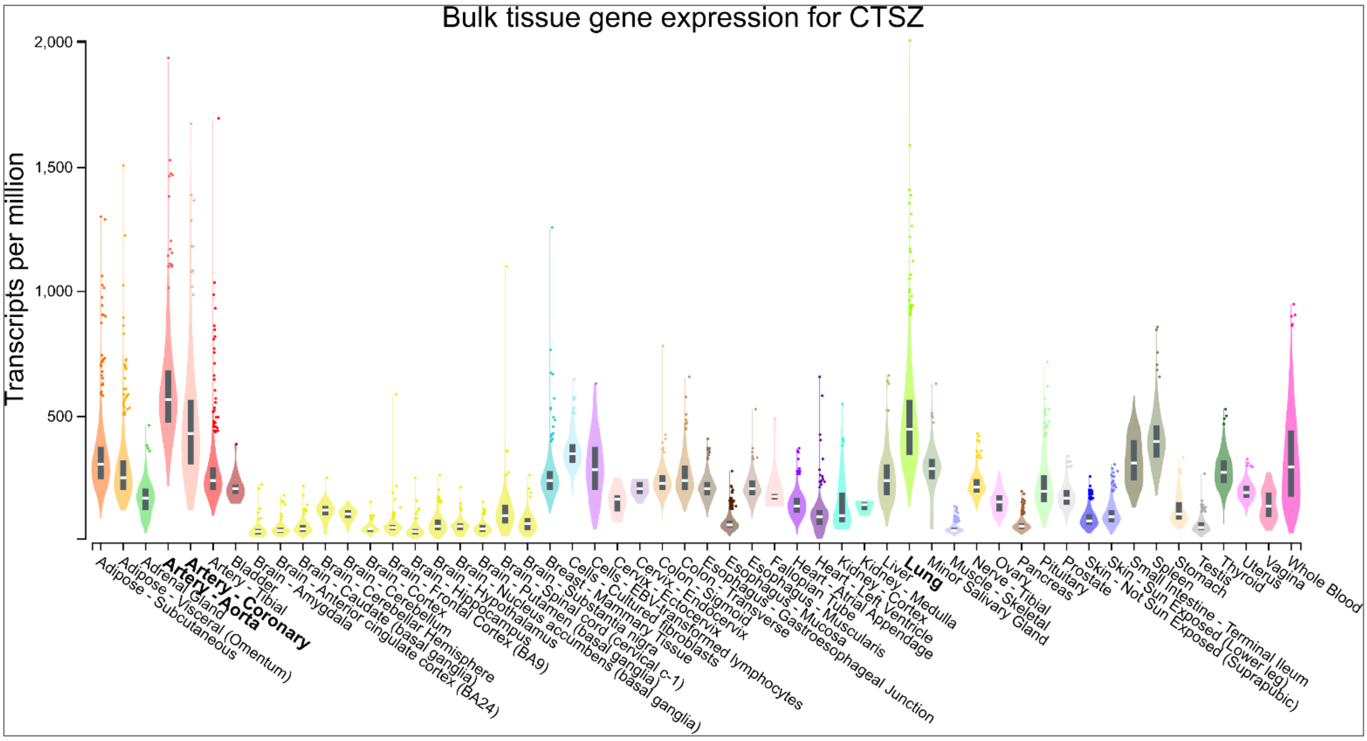

Supplementary Figure 10. Bulk tissue *CTSZ* expression from the GTEx Portal. Each violin depicts the distribution of gene expression values in the tissue site. Top three tissue sites with the highest *CTSZ* expression profiles (“Artery – Aorta”; “Artery – Coronary”; “Lung”) are in bold. GTEx V8 data are plotted.

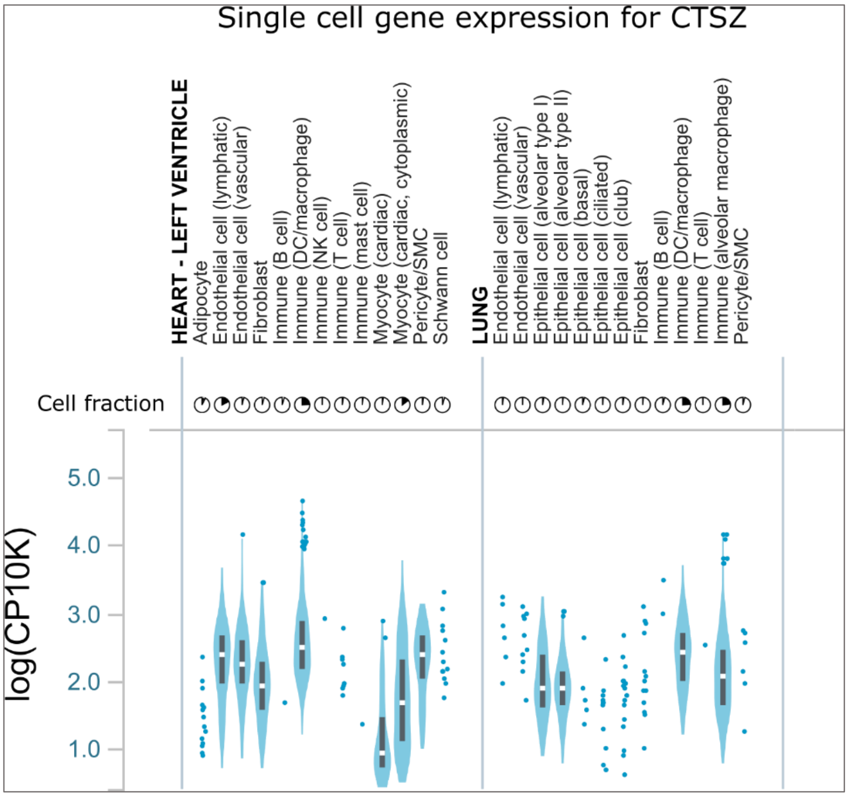

Supplementary Figure 11. Single cell *CTSZ* expression in the heart and the lung from the GTEx Portal. Each violin depicts the distribution of non-zero expression values from individual cells of a cell type from the tissue site. Cell fraction is the fraction of cells in which the gene is detected. CP10K = counts per 10K transcripts. GTEx V8 data are plotted.

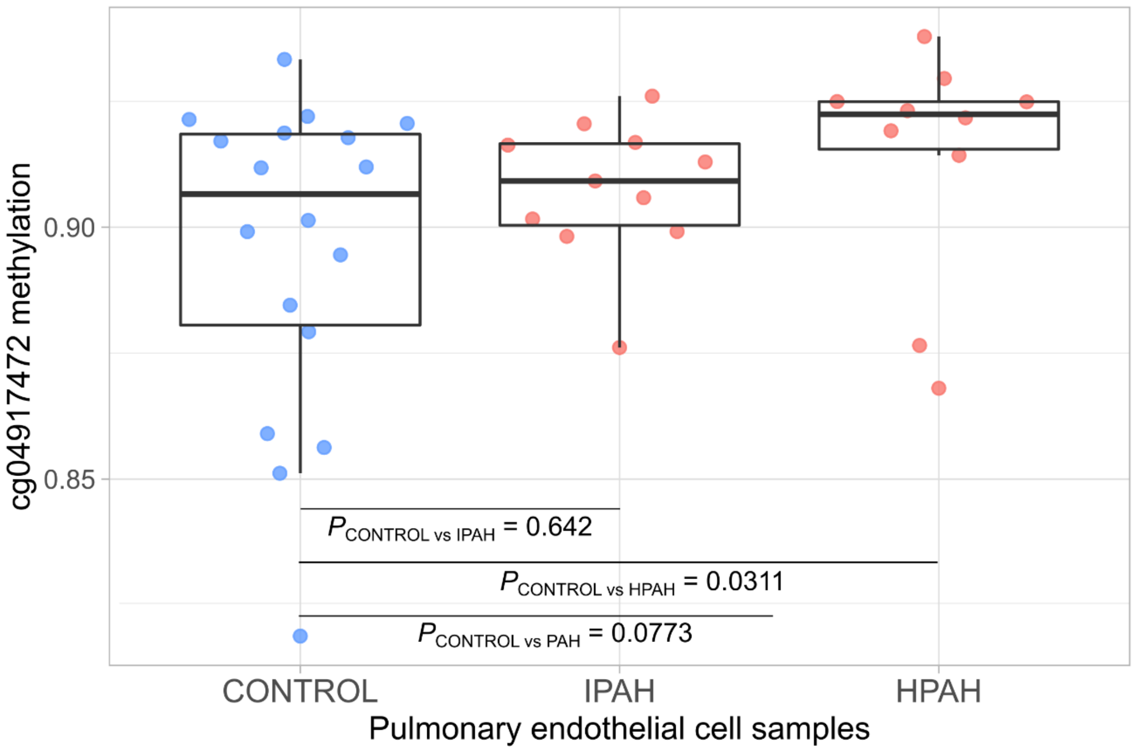

Supplementary Figure 12. CTSZ CpG DNA methylation in pulmonary endothelial cells from controls, heritable PAH (HPAH) and idiopathic PAH (IPAH) [25].

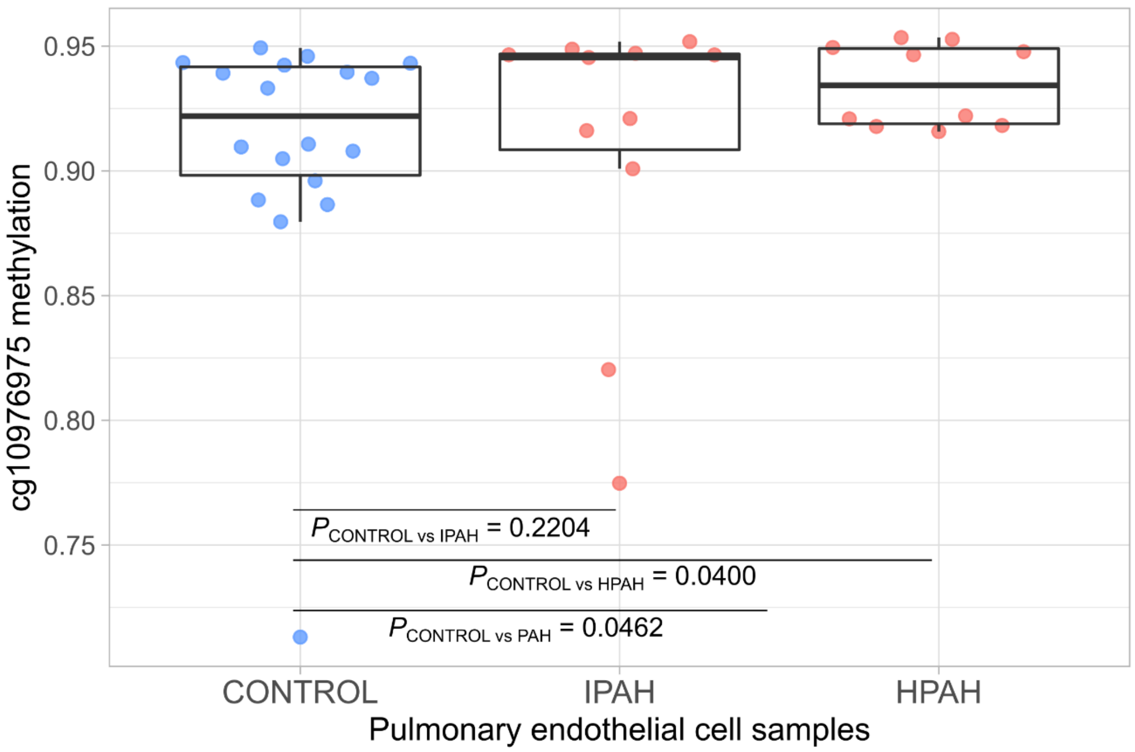

Supplementary Figure 13. BMP10 CpG DNA methylation in pulmonary endothelial cells from controls, heritable PAH (HPAH) and idiopathic PAH (IPAH) [25].

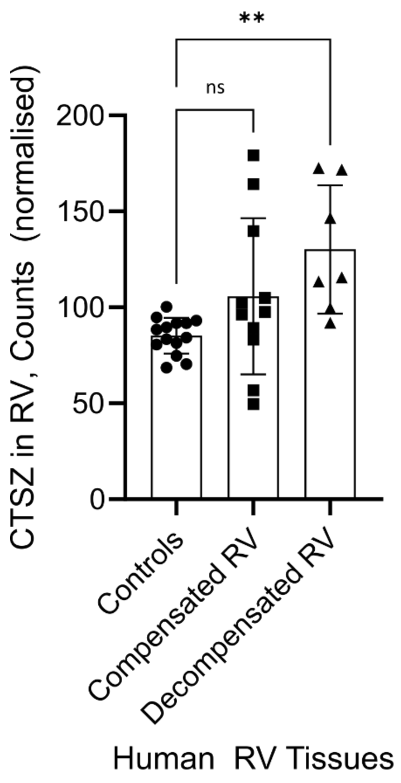

Supplementary Figure 14. CTSZ RNA expression in right ventricle tissue (RV) from non-failing controls (n=14), and patients with compensated (n=11) or decompensated (n=7) RV from public dataset GSE198618. Kruskal-Wallis ANOVA followed by Dunn's multiple comparisons tests versus controls (**=p<0.01, ns = not significant, p>0.05).

UK National PAH Cohort Study Consortium

Marta Bleda, Charaka Hadinnapola, Matthias Haimel, Kate Auckland, Tobias Tilly, Jennifer M. Martin, Katherine Yates, Carmen M. Treacy, Margaret Day, Alan Greenhalgh, Debbie Shipley, Andrew J. Peacock, Val Irvine, Fiona Kennedy, Shahin Moledina, Lynsay MacDonald, Eleni Tamvaki, Anabelle Barnes, Victoria Cookson, Latifa Chentouf, Souad Ali, Shokri Othman, Lavanya Ranganathan, J. Simon R. Gibbs, Rosa DaCosta, Joy Pinguel, Natalie Dormand, Alice Parker, Della Stokes, Dipa Ghedia, Yvonne Tan, Tanaka Ngcozana, Ivy Wanjiku, Gary Polwarth, Rob V. Mackenzie Ross, Jay Suntharalingam, Mark Grover, Ali Kirby, Ali Grove, Katie White, Annette Seatter, Amanda Creaser-Myers, Sara Walker, Stephen Roney, Charles A. Elliot, Athanasios Charalampopoulos, Ian Sabroe, Abdul Hameed, Iain Armstrong, Neil Hamilton, Alex M. K. Rothman, Andrew J. Swift, James M. Wild, Florent Soubrier, Mélanie Eyries, Marc Humbert, David Montani, Barbara Girerd, Laura Scelsi, Stefano Ghio, Henning Gall, Ardi Ghofrani, Richard Trembath, Harm J. Bogaard, Anton Vonk Noordegraaf, Arjan C. Houweling, Anna Huis in't Veld & Gwen Schotte

Freeman Hospital, Newcastle, UK.

Golden Jubilee National Hospital, Glasgow, UK.

Great Ormond Street Hospital, London, UK.

Hammersmith Hospital, London, UK.

Royal Brompton Hospital, London, UK.

Royal Free Hospital, London, UK.

Royal United Bath Hospitals, Bath, UK.

Sheffield NIHR Clinical Research Facility, Royal Hallamshire Hospital, Sheffield, UK.

Département de génétique, hôpital Pitié-Salpêtrière, Assistance Publique-Hôpitaux de Paris, and UMR_S 1166-ICAN, INSERM, UPMC Sorbonne Universités, Paris, France.

Université Paris-Sud, Faculté de Médecine, Université Paris-Saclay, AP-HP, Centre de référence de l'hypertension pulmonaire sévère, INSERM UMR_S 999, Hôpital Bicêtre, Le Kremlin-Bicêtre, France.

San Matteo, Pavia, Italy.

University of Giessen, Giessen, Germany.

VU University Medical Center, Amsterdam, The Netherlands.

For further information please see https://ipahcohort.com/
