# Supplementary material for: Blood DNA methylation profiling identifies cathepsin Z dysregulation in pulmonary arterial hypertension
Source: Nat Commun. 2024 Jan 6;15:330. doi: 10.1038/s41467-023-44683-0 (PMC10771427; doi:10.1038/s41467-023-44683-0)
Supplement: Supplementary file 3 — Description of Additional Supplementary Files [file 41467_2023_44683_MOESM3_ESM.pdf]

Description of Additional Supplementary Files

File Name: Supplementary Data 1
Description: **Study demographics and white blood cell fractions.** Age and white blood cell fractions are presented as median (interquartile range). P-values for between-group differences were derived from Chi-squared tests of independence and one-way ANOVA tests for categorical and continuous variables, respectively. Abbreviations: NK = natural killer. * P-values from two-sided paired T-tests between baseine and follow-up values.

File Name: Supplementary Data 2
Description: **Study demographics, white blood cell fractions and clinical characteristics of baseline and follow-up samples.** Abbreviations: NK = natural killer. P-values were derived from two-sided paired T-tests and McNemar tests for continuous and categorical variables, respectively.

File Name: Supplementary Data 3
Description: : **Top CpG markers associated with PAH reaching a significance threshold of P AH reaching a significance threshold of $P<10^{-5}$.** Three CpG markers reaching the genomic-inflation-factor-adjusted epigenome-wide significance threshold of $P<10^{-7}$ are highlighted. Results are multivariable regression analysis (main EWAS). Effect1 Effect estimate of CpG marker on PAH susceptibility in log(OR) per % increase in methylation. Gene Target gene name from the UCSC database.

File Name: Supplementary Data 4
Description: **Top three CpG marker regions associated with PAH.** CpG markers present in the corresponding regional plot are listed, lead CpG markers are highlighted. Results are multivariable regression analysis (main EWAS). Effect1 Effect estimate of CpG marker on PAH susceptibility in log(OR) per % increase in methylation. Correlation coefficient with lead CpG marker, Spearman`s rank correlation coefficient. P value λ adjusted P-values adjusted for the genomic inflation factor of this study (λ=1.45).

File Name: Supplementary Data 5
Description: **CpG marker associations with PAH at 16 established PAH genes.** CpG marker(s) with Q-values < 0.05 are highlighted. Results are multivariable regression analysis (main EWAS). Effect1 Effect estimate of CpG marker on PAH susceptibility in log(OR) per % increase in methylation. Gene region feature category Gene region feature category describing the CpG position, from UCSC. P-values adjusted for multiple comparisons using the Benjamini & Hochberg method.

File Name: Supplementary Data 6
Description: **Transcript abundance and CpG methylation associations between the top three CpG markers and genes within the CpG-marker-containing TAD (topologically associating domain).** Multivariable regression analysis. Effect2 Effect estimate of the CpG marker on transcript abundance (RNAseq) in beta units per TPM. P value FDR adjusted P-values adjusted for multiple comparisons using the Benjamini & Hochberg method.

File Name: Supplementary Data 7
Description: - **Log10 fold-changes and associated statistics from whole blood RNA comparisons between PAH and healthy controls from UK PAH Cohort study, and correlations with CpG methylation status of epigenetic regulators.** Discovery (n=24 controls versus 120 PAH patients) and validation (another n=24/120) groups are combined for final analysis by edgeR corrected for white blood cells estimated from RNAseq data, age, sex and principal components as described in Rhodes, Otero-Nunez et al AJRCCM 2021. Spearman's Rank correlations performed in all patients with overlapping data.

File Name: Supplementary Data 8
Description: **Correlation of CpG profiles with markers of disease severity.** Rho values and significance of Spearman's rank tests.

File Name: Supplementary Data 9
Description: **Linear regression model for plasma cathepsin Z levels in PAH patients.** Plasma proteins used to predict cathepsin Z levels all measured on SomaLogic SomaScan platform as described in Rhodes et al AJRCCM 2022

File Name: Supplementary Data 10
Description: **Details of individuals who donated lung tissue for immunohistochemistry.**
