# Supplementary material for: Blood DNA methylation profiling identifies cathepsin Z dysregulation in pulmonary arterial hypertension
Source: Nat Commun. 2024 Jan 6;15:330. doi: 10.1038/s41467-023-44683-0 (PMC10771427; doi:10.1038/s41467-023-44683-0)
Supplement: Supplementary file 5 — Reporting Summary [file 41467_2023_44683_MOESM5_ESM.pdf]

nature portfolio

Corresponding author(s): Christopher J Rhodes

Last updated by author(s): Dec 2, 2023

Reporting Summary

Nature Portfolio wishes to improve the reproducibility of the work that we publish. This form provides structure for consistency and transparency in reporting. For further information on Nature Portfolio policies, see our Editorial Policies and the Editorial Policy Checklist.

Statistics

For all statistical analyses, confirm that the following items are present in the figure legend, table legend, main text, or Methods section.

n/a	Confirmed	
☐	☒	The exact sample size (*n*) for each experimental group/condition, given as a discrete number and unit of measurement
☐	☒	A statement on whether measurements were taken from distinct samples or whether the same sample was measured repeatedly
☐	☒	The statistical test(s) used AND whether they are one- or two-sided<br>*Only common tests should be described solely by name; describe more complex techniques in the Methods section.*
☐	☒	A description of all covariates tested
☐	☒	A description of any assumptions or corrections, such as tests of normality and adjustment for multiple comparisons
☐	☒	A full description of the statistical parameters including central tendency (e.g. means) or other basic estimates (e.g. regression coefficient) AND variation (e.g. standard deviation) or associated estimates of uncertainty (e.g. confidence intervals)
☐	☒	For null hypothesis testing, the test statistic (e.g. *F*, *t*, *r*) with confidence intervals, effect sizes, degrees of freedom and *P* value noted<br>*Give P values as exact values whenever suitable.*
☒	☐	For Bayesian analysis, information on the choice of priors and Markov chain Monte Carlo settings
☐	☒	For hierarchical and complex designs, identification of the appropriate level for tests and full reporting of outcomes
☐	☒	Estimates of effect sizes (e.g. Cohen's *d*, Pearson's *r*), indicating how they were calculated

Our web collection on statistics for biologists contains articles on many of the points above.

Software and code

Policy information about availability of computer code

Data collection	None.
Data analysis	We quantile-normalized intensity values for all batches and datasets concatenated together followed by the estimation of white blood cell type sub-populations based on 100 CpG sites by the Houseman method REF18 as implemented in the minfi R package v.1.30.0 REF19. Additionally, we excluded outlying samples based on the top four principal components (PC) of the autosomal, quantile-normalized DNA methylation data, by three times the standard deviation per PC. Ancestry-related principal components were calculated with the EPISTRUCTURE method REF20 implemented in GLINT software v.1.0.4 REF21. Smoking status was predicted using EpiSmokEr: Epigenetic Smoking status Estimator method v.0.1.0 REF22. CpG marker annotations were obtained using the IlluminaHumanMethylationEPICanno R package v.0.6.0 REF23. The code for the CPACOR analysis pipeline was adapted from Lehne et. al. REF17 which was developed and written by Benjamin Lehne (Imperial College London) and Alexander Drong (Oxford University). The code for the low-level quality control was developed and written by Alexander Teumer (University Medicine Greifswald/ Erasmus MC Rotterdam). The code was combined into the current pipeline by Pascal Schlosser and Franziska Grundner-Culemann and it is available at https://github.com/genepi-freiburg/Infinium-preprocessing. The method was then extended to EPIC arrays.

For manuscripts utilizing custom algorithms or software that are central to the research but not yet described in published literature, software must be made available to editors and reviewers. We strongly encourage code deposition in a community repository (e.g. GitHub). See the Nature Portfolio guidelines for submitting code & software for further information.

Data

Policy information about availability of data

All manuscripts must include a data availability statement. This statement should provide the following information, where applicable:

- Accession codes, unique identifiers, or web links for publicly available datasets
- A description of any restrictions on data availability
- For clinical datasets or third party data, please ensure that the statement adheres to our policy

Data from the UK PAH Cohort study are available upon reasonable request to the access committee cohortcoordination@medschl.cam.ac.uk. The use of the Materials or Data must be for research projects and work that falls under the remit of the National Cohort Study of Idiopathic and Heritable PAH or are collaborative projects with one or more of the Partners – see https://www.ipahcohort.com/ for details. The EWAS summary statistics will be deposited at ewascatalog.org upon full publication.
Data used in the preparation of this article were obtained from the Parkinson's Progression Markers Initiative (PPMI) database (www.ppmi-info.org/access-dataspecimens/download-data). For up-to-date information on the study, visit ppmi-info.org. Data used in preparation of this article were obtained from the Alzheimer's Disease Neuroimaging Initiative (ADNI) database (adni.loni.usc.edu). The Northern Finland Birth Cohort data used in this study are available upon collaboration and formal data request only, please see http://www.oulu.fi/nfbc. The GTEx data used for the analyses described in this manuscript were obtained from the GTEx Portal on 05/11/2021.

Research involving human participants, their data, or biological material

Policy information about studies with human participants or human data. See also policy information about sex, gender (identity/presentation), and sexual orientation and race, ethnicity and racism.

Reporting on sex and gender	Individuals of both sexes were analysed and sex is both used as a covariate and a QC parameter to verify correct sample allocation. Sex is given in the summary tables to show distribution across study groups.
Reporting on race, ethnicity, or other socially relevant groupings	We report self-reported ethnicity in the study tables. Ethnicity was not used as a covariate, but is associated with genetic and epigenetic data - the data were analysed with multiple principal components as covariates - Quantile-normalized beta values at each CpG marker were tested for their association with PAH whilst adjusting for sex, age, estimated white blood cell fractions and predicted smoking status, first ten principal components computed from control probes and the first five "epi-structure" principal components to adjust for batch and ancestry differences.
Population characteristics	The UK PAH Cohort Study has a higher proportion of females compared to external control cohorts, in keeping with the female predominance in this disease (Supplementary Table 1). There was good concordance between smoking status predicted from the DNA methylation data and smoking status reported in subsets of the PAH and NFBC1966 cohorts (Supplementary Figure 1). There is significant variance between the UK PAH cohort and the external ADNI and PPMI control groups in terms of proportions of predicted smoking status and four out of six white blood cell fractions (Supplementary Table 1), therefore these were adjusted for in the EWAS model.
Recruitment	Patients and healthy controls are recruited to the UK PAH Cohort study through expert PH centres across the UK. The PH patients are recruited at hospital appointments by specialist research nurses and therefore should accurately reflect the patient population being served by the clinical services. Healthy controls are recruited through a mixture of hospital/ university staff and research centre volunteer programs, which may incur biases associated with volunteering for research whereas the controls included from other published studies are more population-based e.g. NFBC1966 comprises participants from the two northernmost provinces of Finland with expected dates of birth falling in 1966 (n=12,058 births), whereas the ADNI and PPMI are are ongoing observational, international, multicentre (16 US and 5 European sites) studies focussed on cognitive diseases which allowed us to include controls with a range of ages to better match the PH population.
Ethics oversight	The UK PAH Cohort study was approved by East of England Research Ethics Committee [REC] 13/EE/0203.

Note that full information on the approval of the study protocol must also be provided in the manuscript.

Field-specific reporting

Please select the one below that is the best fit for your research. If you are not sure, read the appropriate sections before making your selection.

☒ Life sciences ☐ Behavioural & social sciences ☐ Ecological, evolutionary & environmental sciences

For a reference copy of the document with all sections, see nature.com/documents/nr-reporting-summary-flat.pdf

Life sciences study design

All studies must disclose on these points even when the disclosure is negative.

Sample size	No sample size calculation was performed. The UK PAH Cohort study was powered for genetic research however samples were collected to allow a multitude of studies including this DNA methylation analysis - all available samples were included and represent one of the world's largest PAH patient studies. Controls were included to a roughly 3:1 ratio to patients to maximise power of the study.

Data exclusions	Controls aged over 80 or with a Parkinson's diagnosis > 60 yo, or a diagnosis of Alzheimer's, mild cognitive impairment or unknown diagnosis were excluded to best match age to the PAH population and minimise bias of patient's recruited for cognitive studies.). Staining and hybridisation checks were performed using Illumina's GenomeStudio software v1.0 and were omitted from the CPACOR QC. For each batch (in case of the PAH Cohort Study) and dataset (in the case of external cohorts ADNI, PPMI and NFBC1966) separately, we removed CpG markers with detection p-value>0.049 in over 50% of samples by setting their values to missing for all samples. For each plate and dataset separately, we removed samples with <98% of CpG markers successfully called (detection p-value>0.049). Samples discordant for reported and genetic sex, based on CpGs on the X- and Y-chromosome, were also excluded from the study. We then quantile-normalized intensity values for all batches and datasets concatenated together followed by the estimation of white blood cell type sub-populations based on 100 CpG sites by the Houseman method 18 as implemented in the minfi R package 19. Additionally, we excluded outlying samples based on the top four principal components (PC) of the autosomal, quantile-normalized DNA methylation data, by three times the standard deviation per PC.
Replication	Analyses were repeated with exclusion of distinct control populations and shown to be robust. The association of the lead hit CTSZ with PAH based on DNA methylation analysis was replicated upon analysis of RNA expression data. Cell culture experiments were repeated with independent cultures to a minimum of three independent experiments.
Randomization	No treatment was tested.
Blinding	Technicians who performed the DNA methylation analysis were blinded to the sample status. Blindiing for data and cell culture analysis is not possible but read-outs of assays chosen are quantitative and non-subjective.

Reporting for specific materials, systems and methods

We require information from authors about some types of materials, experimental systems and methods used in many studies. Here, indicate whether each material, system or method listed is relevant to your study. If you are not sure if a list item applies to your research, read the appropriate section before selecting a response.

Materials & experimental systems

n/a	Involved in the study	
☐	☒	Antibodies
☐	☒	Eukaryotic cell lines
☒	☐	Palaeontology and archaeology
☒	☐	Animals and other organisms
☐	☒	Clinical data
☒	☐	Dual use research of concern
☒	☐	Plants

Methods

n/a	Involved in the study	
☒	☐	ChIP-seq
☒	☐	Flow cytometry
☒	☐	MRI-based neuroimaging

Antibodies

Antibodies used	anti-CTSZ antibody (Abcam, UK ab180580) or anti-CD31 (DAKO-MO823/Clone-JC70A) at 1:200 (in DAKO-S2022)
Validation	CTSZ- suitable for WB and IHC-P e.g. Aiba Y et al. Increased expression and altered localization of cathepsin Z are associated with progression to jaundice stage in primary biliary cholangitis. Sci Rep 8:11808 (2018). CD31 Ab widely used - 785 citations have been found for this product.

Eukaryotic cell lines

Policy information about cell lines and Sex and Gender in Research

Cell line source(s)	Human pulmonary artery endothelial cells are obtained from PromoCell GmbH (#C-12241 Lot:#458Z016.14, female) and cultured according to supplier protocols in 10% FBS Endothelial Cell Growth Medium 2 (EGM2; PromoCell #C-22111) at 37°C, 21% O2, and 5% CO2 in a humidified incubator with medium changes every 48 hours. Cells were passaged once they reached 80 to 90% confluence. hPAECs used for experiments were between passages 5 and 8.
Authentication	Vendor authenticate endothelial status of cells using tests showing they are CD31 positive, Dil-Ac-LDL uptake positive.
Mycoplasma contamination	Not tested
Commonly misidentified lines (See ICLAC register)	Not used.

Clinical data

Policy information about clinical studies

All manuscripts should comply with the ICMJE guidelines for publication of clinical research and a completed CONSORT checklist must be included with all submissions.

Clinical trial registration	NCT01907295
Study protocol	www.ipahcohort.com
Data collection	Recruitment at UK expert centres of PH clinics from 19/2/2014 - 24/4/2019
Outcomes	Within the PAH Cohort Study, we implemented the internationally approved diagnostic criteria for idiopathic and heritable PAH, specifically, a raised mean pulmonary artery pressure (mPAP)≥25 mmHg with pulmonary capillary wedge pressure (PCWP)≤15 and pulmonary vascular resistance (PVR)>3 Wood units at rest, and exclusion of known associated diseases REF13.
