# Supplementary figures and images for: Blood DNA methylation profiling identifies cathepsin Z dysregulation in pulmonary arterial hypertension
Source: Nat Commun. 2024 Jan 6;15:330. doi: 10.1038/s41467-023-44683-0 (PMC10771427; doi:10.1038/s41467-023-44683-0)

## Slide 1
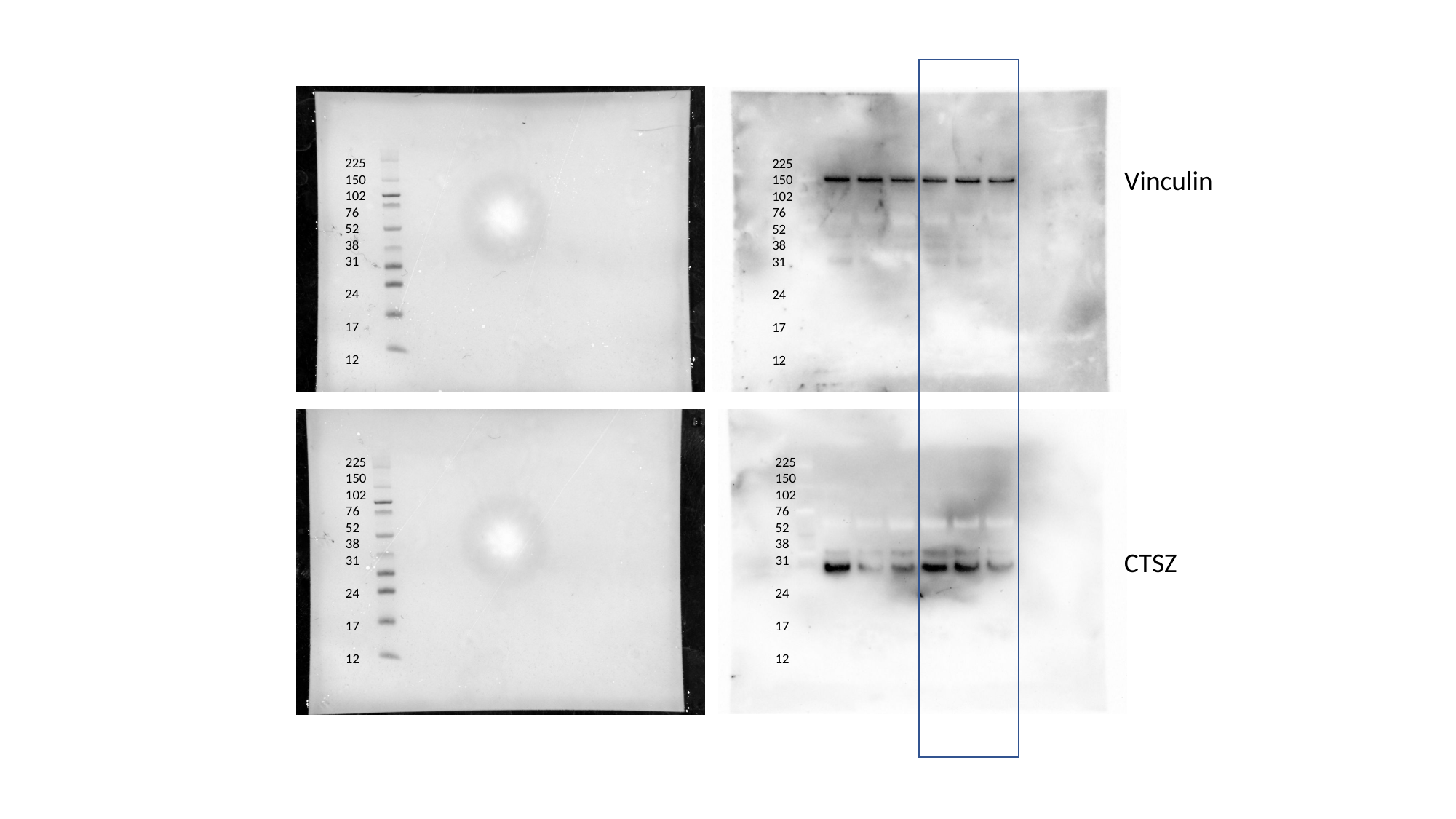

225
150
102
76
52
38
31
24
17
12
225
150
102
76
52
38
31
24
17
12
Vinculin
225
150
102
76
52
38
31
24
17
12
225
150
102
76
52
38
31
24
17
12
CTSZ

Supplement: Supplementary file 6 — Source Data [file 41467_2023_44683_MOESM6_ESM.zip › 426739_2_related_ms_8385647_s53lkf.pptx]
